# New Perspectives on Postmenopausal Osteoporosis: Mechanisms and Potential Therapeutic Strategies of Sirtuins and Oxidative Stress

**DOI:** 10.3390/antiox14050605

**Published:** 2025-05-17

**Authors:** Huiying Zhao, Fan Yu, Wei Wu

**Affiliations:** 1 School of Exercise and Health, Shanghai University of Sports, Shanghai 200438, China; zhaohy1237@163.com (H.Z.); 13734934551@163.com (F.Y.); 2 School of Athletic Performance, Shanghai University of Sports, Shanghai 200438, China

**Keywords:** sirtuins, oxidative stress, postmenopausal osteoporosis, natural activator, natural antioxidant

## Abstract

Estrogen levels are the core factor influencing postmenopausal osteoporosis (PMOP). Estrogen can affect the progression of PMOP by regulating bone metabolism, influencing major signaling pathways related to bone metabolism, and modulating immune responses. When estrogen levels decline, the activity of Sirtuins (SIRTs) is reduced. SIRTs are enzymes that function as NAD+-dependent deacetylases. SIRTs can modulate osteocyte function, sustain mitochondrial homeostasis, and modulate relevant signaling pathways, thereby improving bone metabolic imbalances, reducing bone resorption, and promoting bone formation. In PMOP, SIRT1, SIRT3, and SIRT6 are primarily affected. Oxidative stress (OS) is a crucial factor in PMOP, as it generates excessive reactive oxygen species (ROS) that exacerbate PMOP. There is a certain interplay between SIRTs and OS. The reduced activity of SIRTs leads to intensified OS and the excessive accumulation of ROS. In return, ROS suppresses the AMPK signaling pathway and the synthesis of NAD+, which consequently diminishes the function of SIRTs. Natural SIRT activators and natural antioxidants, which are characterized by high safety, convenience, and minimal side effects, represent a potential therapeutic strategy for PMOP. This study aims to investigate the mechanisms of SIRTs and OS in PMOP and summarize potential therapeutic strategies to assist in the improvement of PMOP.

## 1. Introduction

Postmenopausal osteoporosis (PMOP) is a main osteoporosis intricately linked to the aging process. PMOP usually occurs in postmenopausal women between the ages of 50 and 70 and is a common type of osteoporosis [1]. PMOP is characterized by decreased bone mineral density (BMD), bone microstructural disintegration, bone fragility, and increased fracture risk [2]. In postmenopausal women, there is a significant decrease in estrogen produced by the ovaries, which is the most important factor in PMOP [3]. Decreased estrogen levels contribute to imbalances in bone metabolism, influence major signaling pathways associated with bone metabolism, and regulate the immune system [4]. Complications such as pain and bone deformity caused by the development of PMOP seriously affect the quality of life of postmenopausal women. Hip fractures are among the most severe complications associated with PMOP, typically affecting the femoral neck and intertrochanteric regions. These fractures often lead to extended periods of bed rest and a heightened risk of mortality [5].

Sirtuins (SIRTs) are known as longevity proteins and include SIRT1 to SIRT7 [6]. SIRTs can moderate osteoblast function, maintain mitochondrial homeostasis, and regulate related signaling pathways to ameliorate bone metabolic imbalances, reduce bone resorption, and promote bone formation [7]. When estrogen levels fall, the function of SIRTs is also impacted [8]. Oxidative stress (OS) is a factor in PMOP and has become a major focus of contemporary research. OS inhibits osteoblast (OB) function, promotes osteoclast (OC) production, induces bone mesenchymal stem cell (BMSC) senescence, and interacts with inflammatory responses, leading to an imbalance in bone metabolism and exacerbating PMOP [9]. There is a certain interaction between SIRTs and OS. A decrease in the function of SIRTs causes the excessive generation of reactive oxygen species (ROS), which promotes inflammatory factors and exacerbates OS. OS exacerbation results in diminished SIRT expression and a weakening of the antioxidant capacity, perpetuating a detrimental cycle [10].

The weakening of SIRT function and the exacerbation of OS serve as vital elements of PMOP. The targeted activation of SIRTs and the application of antioxidants have emerged as potential therapeutic strategies to alleviate these two major problems. Compared with synthetic activators and synthetic antioxidants, natural activators and natural antioxidants are more recommended for PMOP patients because of their advantages in terms of side effects, convenience, and safety [11].

In this review, we recap the core pathologic mechanism of PMOP and describe the process by which declining estrogen levels initiate PMOP. The function of SIRTs decreases with decreasing estrogen levels, mainly affecting SIRT1, SIRT3, and SIRT6. At the same time, OS is exacerbated with decreasing estrogen levels. We summarize the roles and mechanisms of SIRTs on PMOP, describe the effects of OS on PMOP, and further illustrate the interaction between SIRT and OS in PMOP. Finally, we conclude with the main natural activators of SIRT1, SIRT3, and SIRT6 and natural antioxidants, aiming to better improve PMOP and enhance life quality.

## 2. Core Mechanisms of Postmenopausal Osteoporosis

Estrogen deficiency is the central cause of PMOP [12]. Estrogen deficiency leads to increased OC activity and the inhibition of OB function [13]. Declining estrogen levels after menopause can impact bone metabolism, key signaling pathways, and the immune system, ultimately leading to PMOP.

### 2.1. Effects of Estrogen on Bone Metabolism

The stability of bone homeostasis hinges on the delicate equilibrium between bone formation and bone resorption, with OB and OC being the key players in this process. Declining estrogen levels after menopause have an impact on the balance of bone metabolism between OB and OC, provoking PMOP [14].

OC is a vital cell responsible for bone resorption, and it belongs to the monocyte-macrophage lineage, which consists of monocyte fusion [15]. OC breaks down the mineralized bone matrix, releasing minerals like calcium and phosphorus into the bloodstream, which in turn regulates the renewal and remodeling of bone tissue [16]. Estrogen has a suppressive effect on OC, and estrogen deficiency affects OC. Estrogen reduces OC production, differentiation, and activity by binding to estrogen receptors (ERs) on the surface of osteoclast precursor cells. When estrogen is deficient, this inhibitory effect is weakened, resulting in an elevated number and enhanced activity of OC, thus accelerating bone resorption and triggering PMOP [17]. The deficiency of estrogen generates an upsurge in the secretion of pro-inflammatory cytokines, subsequently causing an elevated production and activity of OCs [18].

OB is the cell responsible for bone formation, mainly originating from periosteum and BMSCs [19]. BMSCs exhibit the capacity for differentiation into OB. Estrogen facilitates the differentiation of BMSCs into OB, which in turn leads to an increase in OB production. When estrogen is deficient, the number of BMSCs differentiating to OB decreases, and OB production decreases [20] ERs combine with estrogen to regulate OB activity. Estrogen deficiency inhibits OB activity, reduces OB proliferation, decreases bone matrix synthesis, and exacerbates PMOP [21]. Estrogen also has a role in protecting OB from apoptosis, and estrogen deficiency increases OB apoptosis [22].

### 2.2. Effects of Estrogen on OPG-RANKL-RANK and Wnt/β-Catenin Signaling Pathways

OPG-RANKL-RANK and Wnt/β-catenin signaling pathways are two important pathways affecting PMOP. The OPG-RANKL-RANK pathway is mainly associated with the inhibition of OC. The Wnt/β-catenin pathway is mainly associated with the production of OB.

The OPG-RANKL-RANK signaling pathway is a core pathway that regulates bone resorption. Estrogen maintains bone homeostasis through the dual regulation of OPG and RANKL expression [23]. Estrogen inhibits OC production and maintains BMD by up-regulating OPG and down-regulating RANKL. When estrogen is deficient, RANKL expression increases, OPG expression decreases, and the OPG/RANKL ratio decreases [24]. The activity of bone resorption is regulated by the OPG/RANKL ratio. When the OPG/RANKL ratio is high, bone resorption is inhibited; when the OPG/RANKL ratio is low, osteoclast activity is enhanced, and bone resorption increases [25]. The excessive RANKL interacts with RANK on the surface of OC, stimulating their differentiation and activity, and consequently enhancing bone resorption [26]. Bone formation fails to fully counterbalance the increased bone resorption, ultimately resulting in reduced BMD and PMOP. The equilibrium among OPG, RANKL, and RANK is crucial for maintaining BMD [27].

The Wnt/β-catenin signaling pathway plays a crucial role in maintaining bone metabolic homeostasis. It can regulate OB proliferation, differentiation, and function in PMOP [28]. The Dickkopf WNT Signaling Pathway Inhibitor 1 (Dkk1) and Sclerostin (SOST) are inhibitors of the Wnt/β-catenin signaling pathway [29]. After menopause, declining estrogen levels lead to an increased expression of Dkk1 and SOST, which inhibit Wnt signaling and disrupt bone metabolism [30]. Wnt proteins bind the Frizzled/LRP5/6 complex, stabilize β-catenin, and promote its entry into the nucleus, driving the expression of osteogenic differentiation genes such as *Runt-related transcription factor 2 (Runx2)* and Osterix (Osx) [31]. Reduced OB production and function and reduced bone formation occur after a blockade of Wnt signaling [32]. The interaction of DKK1 with LRP5/6 receptors and SOST inhibits Wnt signaling, which work together to weaken LRP5/6-mediated signaling, leading to a shortened half-life of β-catenin, reduced nuclear entry, and the inhibition of *Runx2* and Osx expression [33]. A low expression of *Runx2* and Osx significantly reduces OB and raises PMOP [34].

An interaction between OPG-RANKL-RANK and Wnt/β-catenin pathways also occurs when estrogen levels are decreased. Increased RANKL not only promotes bone resorption but also interacts with LRP5/6 in the Wnt signaling pathway, inhibiting Wnt ligand–receptor binding and decreasing Wnt signaling activity, which in turn reduces bone formation [35]. The inhibition of the Wnt/β-catenin signaling pathway causes decreased OPG and increased RANKL expression, which disrupts the balance of the OPG-RANKL-RANK pathway and exacerbates bone resorption [36]. When estrogen levels decline, the interaction of these two signaling pathways creates a vicious cycle that further exacerbates PMOP.

### 2.3. Effects of Estrogen on Immunity

**Figure 1:**
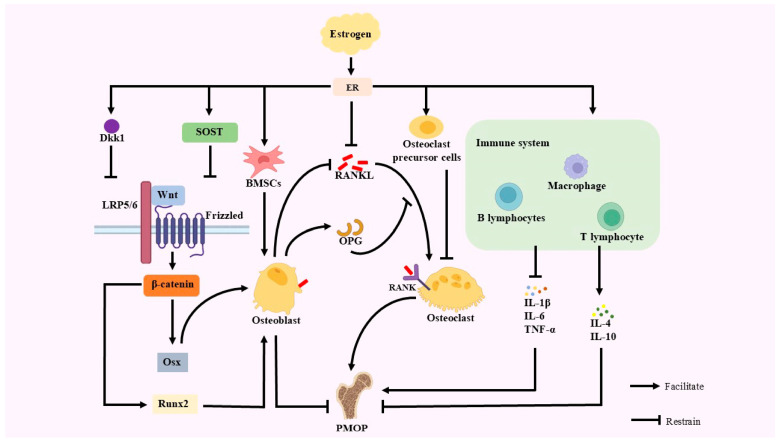
A primary etiological factor for PMOP is estrogen deficiency. When estrogen levels are insufficient, the inhibitory effect of estrogen and OC precursor cells on OC is weakened, leading to increased bone resorption; the number of BMSCs differentiating to OB is reduced, OB production is reduced, and bone synthesis is weakened, leading to bone metabolism. The expression of Dkk1 and SOST, the inhibitors of the Wnt/β-catenin signaling pathway, is decreased when estrogen levels fall. This leads to the inhibition of Wnt proteins through binding to the Frizzled/LRP5/6 complex, which fails to stabilize β-catenin and promote its entry into the nucleus, diminishes the expression of *Runx2* and Osx, and diminishes OB activity. Diminished OB activity promotes decreased OPG expression and increased RANKL expression, which disrupts the OPG-RANKL-RANK pathway balance and exacerbates bone resorption. In estrogen deficiency, estrogen binds to its receptors with the reduced inhibition of RANKL, which in turn leads to the combination of RANKL and RANK on the surface of OC, resulting in increased OC. Estrogen also regulates the immune system. Estrogen inhibits pro-inflammatory factors such as IL-1β, IL-6, and TNF-α, while stimulating anti-inflammatory factors such as IL-4 and IL-10.

Estrogen deficiency triggers alterations in the immune system that subsequently impact bone metabolism and contribute to PMOP [37]. Estrogen inhibits the production of pro-inflammatory factors such as IL-1β (Interleukin-1β, IL-1β), IL-6 (Interleukin-6), and TNF-α (tumor necrosis factor-α, TNF-α), while boosting anti-inflammatory factors such as IL-4 (interleukin 4, IL-4) and IL-10 (interleukin 10, IL-10). When estrogen is lacking, the release of pro-inflammatory factors is increased, stimulating OC formation and activity while inhibiting OB activity, disrupting the bone metabolic balance [38]. In a mouse ovariectomy model, estrogen deficiency was found to lead to the increased production of inflammatory factors, resulting in accelerated bone loss and the exacerbation of PMOP [39]. Estrogen adjusts the activity and function of T cells, B cells, and macrophages, which are immune cells [40]. By binding to ERs on immune cells, estrogen can regulate their proliferation, differentiation, and apoptosis [41]. Estrogen can modulate the synthesis of anti-inflammatory cytokines by immune cells, consequently attenuating inflammation [42]. This regulatory role of estrogen helps maintain the stability of the bone microenvironment. Estrogen deficiency alters the balance of immune cells, increases the activity of pro-inflammatory factors, accelerates bone loss, and exacerbates PMOP [43] (Figure 1).

## 3. Mechanisms of Sirtuins in Postmenopausal Osteoporosis

SIRTs are nicotinamide adenine dinucleotide (NAD+)-dependent histone deacetylases whose activity is highly dependent on NAD+. There are seven members of SIRTs (SIRT1-SIRT7), each of which has a different function in cellular metabolism, OS, inflammatory response, and bone metabolism. When estrogen levels decrease, the activity of SIRTs also decreases, and the functions they originally perform are affected. In the current study, SIRT1, SIRT3, and SIRT6 have been studied more intensively in PMOP, mainly in the three aspects of regulating bone metabolism-related signaling pathways, mitochondrial function and oxidative stress, and inflammatory response.

### 3.1. SIRT1

SIRT1 primarily resides in the nucleus. SIRT1 can mimic the action of estrogen and regulate bone metabolic homeostasis by regulating Estrogen Receptor alpha (ERα) activity, inhibiting OC differentiation, promoting OB differentiation, and decreasing OS, thereby alleviating PMOP [44].

Postmenopausal estrogen levels decline rapidly, causing a significant disequilibrium between bone resorption and bone formation. SIRT1 exerts a protective effect by activating OB differentiation and inhibiting OC activity [45]. In OB, SIRT1 enhances the transcriptional activity of *RUNX2* by deacetylating it and promotes the expression of bone formation-related genes, while inhibiting PPARγ signaling to block the differentiation of BMSCs to adipocytes, thus maintaining OB [46]. In addition, SIRT1 attenuates OS by activating Forkhead box O (FOXO) and peroxisome proliferator-activated receptor gamma coactivator 1-alpha (PGC-1alpha) to reduce OS damage to OB and activate autophagy-related genes to delay cellular senescence [47]. For OC, SIRT1 reduced the RANKL-induced expression of nuclear factor-activated T cell 1 (NFATc1) by blocking its nuclear translocation, owing to the inhibition of the NF-κB signaling pathway. Meanwhile, SIRT1 down-regulated the pro-osteoclastogenic genes tartrate-resistant acid phosphatase (TRAP) and cathepsin K (CTSK) and promoted OPG secretion, thereby inhibiting excessive bone resorption [48]. ERα regulates bone formation and resorption, thus maintaining bone metabolic balance and serving as one of the key factors for ensuring bone health [49]. SIRT1 synergizes with ERα to compensate for estrogen deficiency [50]. In addition, SIRT1 inhibited inflammatory factors such as TNF-α, IL-6, and IL-1β by antagonizing the NF-κB pathway and improved the inflammatory state of the bone microenvironment, whereas its activation of the Wnt/β-catenin pathway further enhanced osteogenic differentiation [51]. SIRT1 activity is affected by estrogen levels, and SIRT1 activity decreases when estrogen declines, which can be ameliorated by activators.

### 3.2. SIRT3

SIRT3 is predominantly located in mitochondria. SIRT3 is involved in the maintenance of bone homeostasis by regulating mitochondrial function, OS, and osteoblast metabolism through deacetylation [52]. After menopause, declining estrogen levels cause mitochondrial dysfunction and ROS accumulation, while SIRT3 mitigates these pathological changes through multiple mechanisms [53].

In OB, SIRT3 significantly reduces mitochondrial ROS by deacetylation and activating superoxide dismutase 2 (SOD2), protecting OB from oxidative damage. SRIT3 reduces ROS while being able to enhance mitochondrial energy metabolism and promote *Runx2* and Osx expression [54]. SIRT3 promotes mitochondrial biosynthesis and enhances OB anabolism by activating the AMPK/PGC-1α signaling axis [55]. For OC, SIRT3 reduced osteoclast precursor cell differentiation to mature OC by suppressing the NF-κB signaling pathway and RANKL-induced ROS burst. Notably, there was a synergistic effect between SIRT3 and SIRT1, which enhanced the antioxidant defense system by co-regulating the FOXO [56]. When estrogen falls, so does SIRT3. SIRT3 activators can compensate for this. Activated SIRT3 can compensate for the effects of estrogen and maintain bone homeostasis.

### 3.3. SIRT6

SIRT6 primarily resides in the nucleus and exerts its effects on OC and OB function mainly by regulating ERα activity, thereby alleviating PMOP.

**Figure 2:**
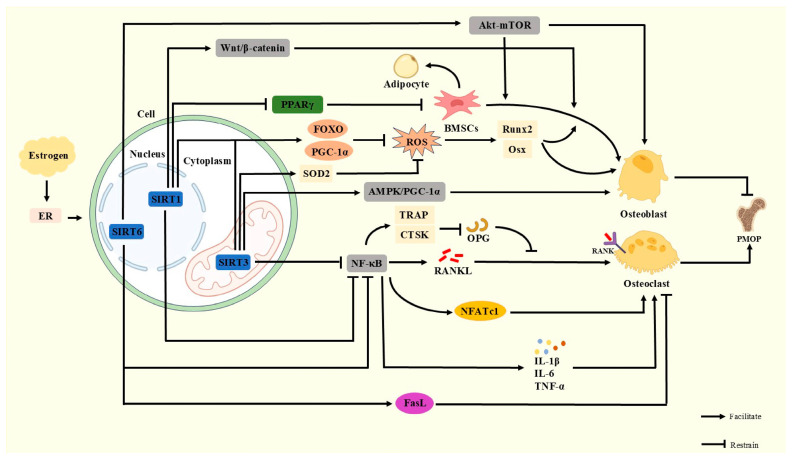
Normally, SIRT1 activates the Wnt/β-catenin pathway to promote bone formation, activates FOXO and PGC-1α to reduce ROS damage to OB, inhibits PPARγ signaling to block BMSCs from differentiating to adipocytes, and inhibits the NF-κB signaling pathway to reduce OC. SIRT3 activates FOXO, PGC-1α, and SOD2, promotes the AMPK/PGC-1α signaling axis, and protects OB, suppresses the NF-κB signaling pathway, and reduces OC. SIRT6 activates the Akt-mTOR pathway and ERα-FasL axis and inhibits the NF-κB signaling pathway to maintain bone metabolic homeostasis. When estrogen declines, the activity of SIRTs decreases and their function is inhibited, leading to the exacerbation of PMOP.

SIRT6 compensates for the negative effects of estrogen deficiency on bone metabolism by combining with ERα and simulating estrogen action [49]. SIRT6 promotes the expression of Factor-related Apoptosis ligand (FasL), induces OC apoptosis, inhibits OC differentiation, and reduces bone resorption [57]. SIRT6 activates the Akt-mTOR pathway, modulating mitochondrial function, promoting the differentiation of BMSCs to OB, and increasing bone formation [58]. When estrogen levels decline, SIRT6 is also affected by it and declines accordingly. Currently, it is possible to activate SIRT6 and restore the function of the ERα-FasL axis to reduce OC production while promoting OB formation. SIRT6 inhibits the inflammatory response caused by declining estrogen levels and mitigates bone destruction [59]. By suppressing the activity of the NF-κB pathway, SIRT6 reduces inflammatory factors, thereby creating a more favorable microenvironment for bone formation [60] (Figure 2).

## 4. Sirtuins and Oxidative Stress

### 4.1. Mechanism of Oxidative Stress in Postmenopausal Osteoporosis

OS is a pathological condition in which there is an overproduction of ROS in the body and an imbalance in the antioxidant system, leading to an imbalance in intracellular redox balance [61]. OS is an important synergistic factor in PMOP production. After menopause, estrogen results in an imbalance of oxidative stress. The reduced activity of SOD2 and glutathione peroxidase (GSH-Px), coupled with the enhanced activity of xanthine oxidase (XOD) and nicotinamide adenine dinucleotide phosphate oxidase (NOX), can lead to the excessive accumulation of ROS in the bone microenvironment and disrupt bone homeostasis [62].

In PMOP, OS exacerbates bone loss through multiple complex mechanisms [63]. After menopause, estrogen levels decline, and the promotion of cellular antioxidant function by estrogen is diminished. Excessive ROS produced by OS activates the NF-κB signaling pathway, up-regulates RANKL, and inhibits OPG, resulting in an elevated RANKL/OPG ratio [64]. This imbalance allows for increased RANKL-RANK binding, accelerated OC differentiation and activation, and enhanced bone resorption [65]. In addition, ROS not only directly stimulates the differentiation of osteoclast precursors into mature OCs but also further affects bone metabolism by inducing the release of inflammatory factors [66]. Increased inflammatory factors promote OC differentiation and inhibit OB function, leading to bone resorption over bone formation and exacerbating PMOP [67].

OS can affect OB through multiple pathways. ROS inhibits *RUNX2* and Osx expression [68]. ROS impairs mitochondrial function, leading to reduced ATP synthesis and oxidative damage to DNA, ultimately inducing OB apoptosis [69]. In addition, ROS suppresses the Wnt/β-catenin signaling pathway, reduces ALP and type I collagen, and weakens the ability of bone matrix synthesis [70]. At the same time, ROS induces the differentiation of BMSCs into adipocytes, resulting in increased bone marrow fat and a reduction in OB numbers [71]. Phospholipid peroxidation is an important process of oxidative stress, and its product, 4-hydroxynonenal (4-HNE), binds to integrin-linked kinase (ILK), leading to the ubiquitinated degradation of ILK. This process, in turn, suppresses *RUNX2* and Osx, thereby impeding the differentiation of OB [72]. In addition, glutathione peroxidase 4 (GPX4) is found in the cytoplasm and mitochondria in OB and safeguards cells against oxidative stress-induced damage. Estrogen regulates the modulation of GPX4. Estrogen deficiency decreases GPX4 expression and increases phospholipid peroxidation in OB, exacerbating PMOP [73].

In this state of chronic oxidative stress, damaged OB and OC interact in a vicious cycle [74]. The impairment of OB leads to decreased bone formation capacity, which is unable to effectively replenish bone loss caused by bone resorption. The over-activation of OC and enhanced bone resorption further damage bone tissues, releasing more ROS and inflammatory factors. Inflammatory factors further damage OB and inhibit its function while promoting OC differentiation and activity [75]. The equilibrium between bone resorption and bone formation can be severely disrupted, leading to increased bone loss and ultimately worsening PMOP.

### 4.2. Interaction Between Sirtuins and Oxidative Stress

SIRTs and OS interact with each other and jointly participate in the pathogenesis and progression of PMOP. A deficiency in estrogen results in decreased NAD+ levels and inhibits SIRT function [76]. The reduced function of SIRTs results in diminished antioxidant capacity and the excessive accumulation of ROS [77]. The excessive accumulation of ROS further weakens the antioxidant capacity and mitochondrial protection of SIRTs by inhibiting AMPK signaling and NAD+ synthesis, while activating the NF-κB signaling pathway and exacerbating OB apoptosis and OC activation [78]. This interaction ultimately brings about reduced bone formation and enhanced bone resorption imbalances.

SIRT1 deacetylates FOXO and PGC-1α, activates antioxidant genes, reduces OS damage to OB, and thus promotes bone formation [79]. When estrogen declines, the decrease in NAD+ leads to a decrease in SIRT1 activity, the inhibition of FOXO and PGC-1α function, diminished antioxidant capacity, increased ROS, and exacerbated OS [80]. An animal study found that a single underdose of SIRT1 resulted in a significant decrease in BMD among adult female mice, suggesting a protective role for SIRT1 in PMOP [81]. SIRT3 regulates mitochondrial homeostasis and antioxidant enzyme activity. SIRT1 can activate FOXO and PGC-1α, as well as SIRT3, to reduce the overproduction of ROS. SIRT3 also activates SOD2 via deacetylation to inhibit ROS production [82]. SIRT3 regulates mitochondrial homeostasis and antioxidant enzyme activity. SIRT1 can activate FOXO and PGC-1α, as well as SIRT3, to reduce the overproduction of ROS. SIRT3 also activates SOD2 through deacetylation to inhibit ROS generation [83]. When estrogen declines, the impaired SIRT3 function leads to a decreased activity of SOD2 and the accumulation of ROS in the mitochondria, which ultimately triggers an exacerbation of OS. SIRT6 can activate the SIRT6-ERα-FasL axis, promote OC apoptosis, reduce bone resorption, and alleviate the damage of OS to postmenopausal osteoporosis [84]. Deficient estrogen leads to decreased expression levels of the SIRT6-ERα-FasL axis and exacerbates OS [85]. In addition, SIRT6 inhibits the NF-κB signaling pathway, thereby reducing the release of inflammatory factors and alleviating the destruction of the bone microenvironment by ROS [86]. When estrogen levels decline, the ability of SIRT6 to inhibit inflammation is diminished, exacerbating oxidative stress.

**Figure 3:**
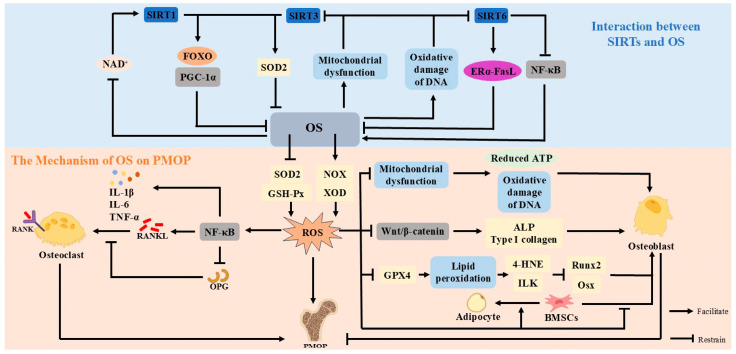
OS leads to the overproduction of ROS. Excessive ROS will inhibit the Wnt/β-catenin pathway, mitochondrial function and GPX4, and phospholipid peroxidation, leading to OB damage; increased ROS can trigger the NF-κB signaling pathway, affect the OPG-RANKL-RANK signaling pathway, and promote the activity of OC, which will ultimately lead to PMOP. SIRTs and OS exhibit a reciprocal interaction. SIRT1, SIRT3, and SIRT6 can attenuate OS by activating the expression of antioxidant genes and the ERα-FasL axis. The inhibitory effect of SIRTs on OS is weakened when estrogen is deficient, affecting bone metabolic homeostasis. The exacerbation of OS affects the activity of SIRTs by inhibiting NAD+, impairing mitochondrial function, and oxidatively damaging DNA to make the expression of SIRT1, SIRT3, and SIRT6 decrease.

In PMOP, the exacerbation of OS leads to a decreased expression of SIRT1, SIRT3, and SIRT6 through multiple mechanisms. OS leads to increased intracellular ROS levels, which deplete NAD⁺ and directly inhibit NAD⁺-dependent SIRT1 activity [87]. OS-induced mitochondrial dysfunction inhibits SIRT3 and reduces SOD2, thereby impairing OB activity [88]. In addition, OS inhibits the expression of SIRT6 by oxidative damage to DNA and affects the NF-κB signaling pathway, resulting in an increase in inflammatory factors, exacerbating OC activity and inhibiting OB differentiation [89]. The combined effect of these mechanisms causes an imbalance in bone metabolism that exacerbates PMOP (Figure 3).

## 5. Targeted Activation of Sirtuins and Potential Therapeutic Strategies Against Oxidative Stress

With the decline in estrogen levels, both the decrease in SIRTs activity and the aggravation of OS will further exacerbate PMOP. In recent years, natural SIRT activators and natural antioxidants have gradually become a research hotspot due to their high safety, convenience, and minimal side effects [90]. These natural compounds can not only regulate the balance of bone metabolism by activating SIRTs but also protect osteoblasts by scavenging free radicals and inhibiting OS, which shows a promising application in PMOP [91].

### 5.1. Natural Activators of Sirtuins

In recent years, the natural activators of SIRTs have received much attention for their potential therapeutic value in metabolic diseases such as osteoporosis. The activators of the SIRTs family, especially SIRT1, SIRT3, and SIRT6, have been demonstrated to have significant effects in improving cellular energy metabolism, reducing OS, and modulating the inflammatory response. Activators are categorized into natural and synthetic activators. Natural activators are widely available, mainly from daily food, and are easy to obtain. In addition, natural activators are generally safer and have fewer side effects, making them friendlier to the human body and suitable for long-term use.

The natural activators of SIRT1 mainly include resveratrol, pterostilbene, curcumin, quercetin, and genistein. They act on PMOP by activating SIRT1 through a variety of mechanisms. Resveratrol, a polyphenolic compound that occurs naturally, is widely found in grapes, red wine, blueberries, strawberries, peanuts, and mulberries [92]. Resveratrol directly binds to SIRT1, enhances the deacetylase activity of SIRT1, and activates SIRT1. Activated SIRT1 enhances the antioxidant capacity of osteoblasts, reduces OS, and improves PMOP [93]. A study found that regular resveratrol supplementation significantly increased BMD in the lumbar spine and femoral neck of postmenopausal women [94]. Resveratrol also regulates cellular energy metabolism and promotes mitochondrial function through the SIRT1/PGC1α axis, which further maintains the normal physiological function of osteoblasts [95]. Pterostilbene is a naturally occurring resveratrol derivative with enhanced antioxidant capacity, primarily derived from grapes, blueberries, cranberries, and peanuts [96]. By binding to the active site of SIRT1, pterostilbene enhances its deacetylase activity [97]. Similarly to resveratrol, pterostilbene-activated SIRT1 regulates cellular metabolism and antioxidant defense mechanisms, reduces OS damage to bone, and exerts a therapeutic effect on PMOP [98]. Curcumin, a natural compound extracted from turmeric and found mainly in turmeric, curry, and mustard, has antioxidant and anti-inflammatory properties [99]. Curcumin can directly bind to SIRT1 and enhance its activity [100]. Curcumin, by activating the Nrf2 signaling pathway, further enhances SIRT1 activity [101]. The activated SIRT1 and Nrf2 signaling pathways protect OB from oxidative stress damage and promote osteoblast proliferation and differentiation [102]. Quercetin is a flavonoid found primarily in onions, kale, blueberries, cranberries, and red wine [103]. Quercetin regulates cellular autophagy and reduces osteoblast apoptosis by activating SIRT1 activity [104]. Quercetin also reduces OS and improves PMOP through SIRT1-mediated antioxidant actions [105]. Genistein is a phytoestrogen with estrogen-like bioactivity found mainly in soybeans, tofu, soymilk, and Pueraria [106]. Genistein simulates the role of estrogen, activates SIRT1, and regulates bone metabolism-related signaling pathways [107]. Genistein-activated SIRT1 regulates mitochondrial function, reduces OS, and exerts protective effects on osteoblasts [108].

The natural activators of SIRT3 mainly include resveratrol, curcumin, and honokiol. These natural activators of SIRT3 exert cellular protective effects mainly by regulating mitochondrial metabolism and reducing OS. Resveratrol up-regulates SOD2 expression by activating the Sirt3/FOXO3a pathway to reduce ROS accumulation and mitigate cellular damage from oxidative stress [109]. In addition, resveratrol also regulates mitochondrial function through SIRT3, which improves mitochondrial metabolism and enhances cellular energy metabolism efficiency [110]. Curcumin activates SIRT3, reduces SOD2 and ROS, and inhibits excessive cellular autophagy [111]. In addition, curcumin regulates mitochondrial metabolism through SIRT3 and improves cellular energy status [112]. Honokiol is a natural compound extracted from Magnolia officinalis with anti-inflammatory and antioxidant properties [113]. Honokiol, by restraining the NF-κB pathway, attenuates the inflammatory response. Meanwhile, Honokiol activated SIRT3 by regulating mitochondrial function [114]. Activated SIRT3 reduces OS and protects cells from damage [115].

The main natural activators of SIRT6 include luteolin, quercetin, anthocyanins, and catalpol. These natural activators have been shown to bind directly to the active region of SIRT6, enhancing its catalytic activity, reducing OS, and inhibiting inflammatory responses. Luteolin and quercetin are both flavonoids that are widely found in plants [116]. Luteolin is mainly derived from celery, green peppers, red peppers, broccoli, and spinach [117]. Luteolin and quercetin activate Sirt6 by binding to its active region [118]. Activated SIRT6 reduces the production of the senescence-associated secretory phenotype (SASP), inhibits the activation of the NF-κB signaling pathway, reduces inflammatory factors, delays cellular senescence, and decelerates OS [119]. In addition, luteolin and quercetin regulate cellular metabolism and antioxidant- and inflammation-related gene expression through SIRT6 [120]. Anthocyanins are found in blueberries, strawberries, mulberries, blackberries, grapes, and blackcurrants [121]. Anthocyanins bind to SIRT6 and enhance its catalytic activity to regulate glycolysis and energy metabolism [122]. Activated SIRT6 reduces ROS accumulation, regulates mitochondrial function, and mitigates oxidative damage [123]. Moreover, anthocyanins can inhibit the NF-κB signaling pathway, which helps to mitigate the adverse effects of inflammatory factors on bone cells [124]. Catalpol is a herbal ingredient derived from the Rehmanniae Radix [125]. Catalpol has been shown to significantly alleviate osteoporosis symptoms in ovariectomized rats through the activation of the SIRT6-ERα-FasL axis [84]. Catalpol enhances SIRT6 activity, regulates cellular metabolism, impedes the NF-κB signaling pathway, attenuates inflammatory responses, and diminishes ROS generation [126].

Natural activators of SIRT1, SIRT3, and SIRT6 are detailed in Table 1.

### 5.2. Natural Antioxidants

OS is an important causative factor in PMOP. After menopause, lower estrogen levels contribute to a weakening of antioxidant capacity and an increase in OS, affecting bone metabolic homeostasis [127]. Antioxidants can mitigate the harm caused by OS directly by reacting with free radicals, or indirectly by inhibiting the activity of free radical-producing enzymes, or increasing the activity of intracellular antioxidant enzymes [128]. Natural antioxidants, which are widely available, relatively safe, and have few side effects, can improve bone metabolism through a variety of mechanisms [129]. The application of natural antioxidants in PMOP has received widespread attention and may provide new ideas for the treatment of PMOP.

Resveratrol, curcumin, anthocyanins, and pterostilbene are common polyphenolic compounds known for their ability to neutralize free radicals and reduce inflammation, supporting bone metabolism [130]. The antioxidant capacity of resveratrol can scavenge free radicals and reduce OS damage, thus maintaining bone metabolism [131]. Curcumin has the same antioxidant and anti-inflammatory properties. Curcumin and resveratrol can reduce the production of inflammatory factors by synergistically inhibiting the NF-κB pathway, increasing the up-regulation of the *RUNX2* gene and decreasing the activity of OC, which in turn reduces bone resorption [132]. Anthocyanins can regulate the RANKL pathway to inhibit OS, suppress OC differentiation, reduce OC production, and maintain the balance of bone metabolism [133]. Pterostilbene is isolated from Pterocarpus marsupium. Pterostilbene acts as a natural antioxidant that can protect cells from oxidative damage by scavenging free radicals and activating the Nrf2 pathway, reducing OS [134]. Pterostilbene induces OB activity, enhances bone formation, and has excellent osteoprotective effects against bone loss caused by estrogen deficiency [135].

Vitamin E, coenzyme Q10, and lycopene, as common fat-soluble antioxidants, are able to reduce OS damage to bone cells. Vitamin E is mainly derived from wheat germ oil, sunflower oil, corn oil, peanut oil, almond, hazelnuts, and cashew nuts [136]. Vitamin E, capable of penetrating the lipid bilayer of cell membranes to provide protection against lipid peroxidation and maintain membrane integrity, has been shown to effectively improve BMD and bone microarchitecture when supplemented [137]. Vitamin E intake has a positive correlation with BMD, and higher intakes are linked to a lower risk of fractures [138]. Vitamin E has several isomers. In vitamin E, α-Tocopherol is the predominant form. By neutralizing ROS and free radicals in the body, it helps protect cells from oxidative damage [139]. Coenzyme Q10 is mainly derived from pig heart, pig liver, chicken liver, beef, pork, and chicken [140]. Coenzyme Q10 activates the Nrf2 signaling pathway and relieves OS [141]. Coenzyme Q10 can also maintain bone metabolism by regulating mitochondrial function, reducing ROS, and protecting osteoblasts from oxidative damage [142]. Lycopene is widely found, mainly in tomatoes, watermelon, grapefruit, and carrots, and is a highly effective antioxidant [143]. Lycopene plays an active role in PMOP by reducing OS through its powerful antioxidant capacity [144]. Lycopene inhibits OC activity and reduces bone resorption; it also promotes OB proliferation and differentiation, which in turn increases bone formation [145]. Vitamin C serves as a water-soluble antioxidant, primarily acting within the aqueous cytoplasm, scavenging ROS and preventing oxidative damage to intracellular components [146]. Vitamin C is mainly derived from lemons, limes, grapefruit, oranges, strawberries, and raspberries [147]. Adequate vitamin C supplementation can safeguard bone cells against oxidative damage through the elimination of free radicals and the suppression of OS [148].

Natural antioxidants of SIRT1, SIRT3, and SIRT6 are detailed in Table 2.

## 6. Conclusions and Perspectives

Decreased estrogen levels are the main contributor to PMOP. Estrogen can influence the course of PMOP through bone metabolism, related important signaling pathways, and immune regulation. This review focuses on the changes in estrogen levels and explores the effects of SIRT and OS on PMOP. When estrogen levels decrease, the activity of SIRTs also decreases. Within the SIRT family, SIRT1, SIRT3, and SIRT6 contribute to maintaining bone metabolism. They can inhibit OC and promote OB by regulating bone metabolism-related signaling pathways, regulate mitochondrial function to mitigate the damage caused by OS, and inhibit inflammatory factors to control the inflammatory response. OS is an important part of the PMOP disease process. OS causes the excessive accumulation of ROS, leading to an imbalance in bone homeostasis and exacerbating PMOP. There is a certain interaction between SIRTs and OS. The reduced activity of SIRTs leads to the aggravation of OS and the excessive accumulation of ROS. Excessive ROS generated by OS in turn weakens the function of SIRTs by restricting the AMPK signaling pathway and the synthesis of NAD+, activates the NF-κB signaling pathway, and disrupts bone metabolism.

The activation of SIRTs and the mitigation of OS has become an approach to treat PMOP. Natural activators and antioxidants are safer and more convenient and produce far fewer side effects than synthetic activators and antioxidants, making them more acceptable. The natural activators of SIRTs and natural antioxidants can activate SIRT activity to regulate bone metabolism and inhibit OS to protect osteoblasts, which shows a promising application in the treatment of PMOP.

SIRT1, SIRT3, and SIRT6 have been extensively studied in PMOP, and this review provides a summary of these studies. Other members of the SIRT family, such as SIRT2, SIRT4, SIRT5, and SIRT7, may also indirectly affect bone metabolism by regulating metabolism and inflammatory responses. However, due to the limited number of studies, this review does not elaborate on these aspects. To facilitate the discovery of new therapeutic targets, more detailed studies and in-depth discussions will be required in the future. In addition, the specific optimal dosages of these natural activators and natural antioxidants need to be studied and refined even further so that the improvement of PMOP can be further advanced.

## Figures and Tables

**Table 1:** Natural activators of SIRT1, SIRT3, and SIRT6.

Sirtuins	Natural Activator	Sources	Biological Effects	Ref.
SIRT1	Resveratrol	Grapes, red wine, blueberries, strawberries, peanuts, and mulberries	Reduce oxidative stress, regulate the SIRT1/PGC1α axis, and promote mitochondrial function	[92,93,94,95]
	Pterostilbene	Grapes, blueberries, cranberries, and peanuts	Regulate cell metabolism; reduce oxidative stress	[96,97,98]
	Curcumin	Turmeric, curry, and mustard	Activate the Nrf2 signaling pathway and reduce oxidative stress	[99,100,101,102]
	Quercetin	Onions, kale, blueberries, cranberries, and red wine	Regulate autophagy and reduce apoptosis of bone cells; reduce oxidative stress	[103,104,105]
	Genistein	Soy, tofu, soy milk, and Pueraria	Mimic the role of estrogen and regulates bone metabolism; regulate cell metabolism	[106,107,108]
SIRT3	Resveratrol	Grapes, red wine, blueberries, strawberries, peanuts, and mulberries	Activate Sirt3/FoxO3a pathway, up-regulate the expression of SOD2, and reduce the accumulation of ROS	[109,110]
	Curcumin	Turmeric, curry, and mustard	Inhibit the NF-κB signaling pathway; Rrgulate mitochondrial metabolism	[111,112]
	Honokiol	Magnolia officinalis	Restrains NF-κB signaling pathway; promotes mitochondrial function	[113,114,115]
SIRT6	Luteolin	Celery, green pepper, red pepper, broccoli, and spinach	Inhibit NF-κB signaling pathway; reduce oxidative stress	[116,117,118,119,120]
	Quercetin	Onions, kale, blueberries, cranberries, and red wine	Inhibit NF-κB signaling pathway; reduce oxidative stress	[116,118,119,120]
	Anthocyanins	Blueberries, strawberries, mulberries, blackberries, grapes, and blackcurrants	Regulate glycolysis and energy metabolism; regulate mitochondrial metabolism	[121,122,123,124]
	Catalpol	Rehmanniae Radix	Regulate cell metabolism; inhibit the NF-κB signaling pathway, alleviate inflammatory response, and reduce oxidative stress	[125,126,127]

**Table 2:** Natural antioxidants of SIRT1, SIRT3, and SIRT6.

Natural Antioxidant		Sources	Biological Effects	Ref.
Polyphenols	Resveratrol	Grapes, red wine, blueberries, strawberries, peanuts, and mulberries	Scavenge free radicals; inhibit NF-κB pathway; increase the up-regulation of the *RUNX2* gene; reduce the activity of osteoclasts; reduce oxidative stress	[132,133]
	Curcumin	Turmeric, curry, and mustard	Inhibit NF-κB pathway; increase the up-regulation of the *RUNX2* gene; reduce the activity of osteoclasts	[133]
	Anthocyanins	Blueberries, strawberries, mulberries, blackberries, grapes, and blackcurrants	Regulate the RANKL pathway; inhibit oxidative stress; maintain the balance of bone metabolism	[134]
	Pterostilbene	Grapes, blueberries, cranberries, and peanuts	Scavenge free radicals; activate the Nrf2 pathway; induce osteogenic activity	[135,136]
Fat-soluble antioxidant	Vitamin E	Wheat germ oil, sunflower oil, corn oil, peanut oil, almond, hazelnut, and cashew nut	Improve bone density and bone microstructure; scavenge ROS and free radicals	[137,138,139,140]
	Coenzyme Q10	Pig heart, pig liver, chicken liver, beef, pork, and chicken	Activate the Nrf2 signaling pathway; regulate mitochondrial function	[141,142,143]
	Lycopene	Tomatoes, watermelon, grapefruit, and carrots	Inhibit osteoclasts and promote osteoblasts	[144,145,146]
Water-soluble antioxidant	Vitamin C	Lemons, limes, grapefruit, oranges, strawberries, and raspberries	Scavenge free radicals and inhibit oxidative stress	[147,148]

## Data Availability

No new data were created or analyzed in this study. Data sharing is not applicable to this article.

## Abbreviations

The following abbreviations are used in this manuscript:

PMOP	postmenopausal osteoporosis
SIRTs	Sirtuins
NAD+	nicotinamide adenine dinucleotide
OS	oxidative stress
ROS	reactive oxygen species
BMD	bone mineral density
OB	osteoblast
OC	osteoclast
BMSCs	bone mesenchymal stem cells
ERs	estrogen receptors
Dkk1	Inhibitor 1
SOST	Sclerostin
*Runx2*	*Runt-related transcription factor 2*
Osx	Osterix
IL-1β	Interleukin-1β
IL-6	Interleukin-6
TNF-α	tumor necrosis factor-α
IL-4	interleukin 4
IL-10	interleukin 10
NAD+	Nicotinamide adenine dinucleotide
ERα	Estrogen Receptor alpha
FOXO	Forkhead box O
PGC-1α	peroxisome proliferator-activated receptor γ coactivator 1-alpha
NFATc1	nuclear factor-activated T cell 1
TRAP	tartrate-resistant acid phosphatase
CTSK	cathepsin K
SOD2	superoxide dismutase 2
FasL	Factor-related Apoptosis ligand
GSH-Px	glutathione peroxidase
NOX	reduced nicotinamide adenine dinucleotide phosphate oxidase
XOD	xanthine oxidase
4-HNE	4-hydroxynonenal
ILK	integrin-linked kinase
GPX4	glutathione peroxidase 4
